# Bibliometrics approach to evaluating the research impact of CTSAs: A pilot study

**DOI:** 10.1017/cts.2020.29

**Published:** 2020-04-02

**Authors:** Fei Yu, Allison Alicia Van, Tanha Patel, Nandita Mani, Andrea Carnegie, Giselle M. Corbie-Smith, Timothy Carey, John Buse, Gaurav Dave

**Affiliations:** 1 Health Sciences Library, University of North Carolina at Chapel Hill, Chapel Hill, NC, USA; 2 North Carolina Translational and Clinical Institute, University of North Carolina at Chapel Hill, Chapel Hill, NC, USA; 3 School of Medicine, University of North Carolina at Chapel Hill, Chapel Hill, NC, USA

**Keywords:** CTSA evaluation, translational research, bibliometrics, network analysis, research impact measurement

## Abstract

**Introduction:**

To enhance the performance evaluation of Clinical and Translational Science Award (CTSA) hubs, we examined the utility of advanced bibliometric measures that go beyond simple publication counts to demonstrate the impact of translational research output.

**Methods:**

The sampled data included North Carolina Translational and Clinical Science Institute (NC TraCS)-supported publications produced between September 2008 and March 2017. We adopted advanced bibliometric measures and a state-of-the-art bibliometric network analysis tool to assess research productivity, citation impact, the scope of research collaboration, and the clusters of research topics.

**Results:**

Totally, 754 NC TraCS-supported publications generated over 24,000 citation counts by April 2017 with an average of 33 cites per article. NC TraCS-supported research papers received more than twice as many cites per year as the average National Institute of Health-funded research publications from the same field and time. We identified the top productive researchers and their networks within the CTSA hub. Findings demonstrated the impact of NC TraCS in facilitating interdisciplinary collaborations within the CTSA hub and across the CTSA consortium and connecting researchers with right peers and organizations.

**Conclusion:**

Both improved bibliometrics measures and bibliometric network analysis can bring new perspectives to CTSA evaluation via citation influence and the scope of research collaborations.

## Introduction

In October 2006, the National Institutes of Health (NIH) launched the Clinical and Translational Science Award (CTSA) to accelerate the translation of scientific discoveries into practical applications to improve human health [1]. The National Center for Advancing Translational Sciences (NCATS) at NIH has since funded and supported CTSA hubs at more than 50 medical research institutes across the United States [2]. These CTSA hubs engage in a wide variety of institutional activities, all aiming to increase the quality, transparency, translation, and reproducibility of scientific research and to “get more treatments to more patients more quickly” [3]. At each CTSA hub, evaluation is conducted to assess its activities, outcomes, and impact on the translational research enterprise.

Evaluation in this context relies on a diverse set of process and outcome metrics meant to capture a broad range of activities and dimensions of successful translational science [4]. These activities may include effective training of translational scientists; development of tools and methods that open new fields of inquiry; clinical trials support services to increase efficiency and start-ups; research support of collaboration; and the impact and influence of supported researchers [3]. One such evaluation dimension of translational and clinical sciences is to measure research productivity and collaboration impact [5]. For example, do CTSAs foster intra- and inter-institutional networking and research collaborations? Do CTSAs enhance scholarly productivity of investigators within and outside of their institution? To answer these and other evaluative questions, we need additional tools and methods that improve the current CTSA evaluation strategy in assessing translational research impact of a hub.

Bibliometrics is the quantitative analysis of publication information and is identified as a core method embedded in conceptual frameworks and models that assess research impact in health sciences research domains [6,7]. Some of the basic measures in bibliometrics like the number of publications and the citation count (i.e., total number of times a published article was cited by other papers) are established components of CTSA evaluation. CTSA hubs annually report the number of publications supported by their hubs as a measure of research productivity and performance [8,9]. As an important bibliometrics measure, citation impact is often used to indicate the influence of research output.

Researchers and institutions have long been adopting basic measures including citation counts, H-index [10], and journal impact factor to demonstrate their research influence. However, citation analysis can be biased and controversial if used as a primary or singular method of evaluating research productivity and performance. For example, citation counts do not reflect work cited for flaws or inaccuracy in the study [11]. The calculation of H-index disadvantages early-stage researchers by not considering the highly cited works or total citation counts [10]. The journal impact factor is a “short-term index” based on citation counts during two or three years and averages citation counts for all articles in a journal [12]. Citation impact measures also depend on several other factors, such as research field, type of journals, article type, publication year, and the scope of journal articles indexed by citation-tracking databases [10,13].

The recent advancements of bibliometrics resources and information technology allow us to shift from basic bibliometrics (process-level measures) such as publications or citation counts to field- and time-normalized “comparative” approaches (impact-level measures) such as snowball metrics, benchmarking, and outputs in top percentiles [14,15]. Research-supporting institutions are now able to gain a more nuanced picture of the citation impact of research than can be captured in raw citation counts alone [4,16]. One of CTSA consortium-led evaluation workgroups has explored the feasibility and utility of both basic and improved bibliometric approaches. Workgroup members concluded that bibliometric analysis contributes to the evaluation of CTSA-supported clinical and translational research as an important complementary method [4,17].

Emerging as a strong interest among CTSAs, bibliometrics also prompted CTSA hubs to collaborate with librarians at their institutional health sciences or medical libraries for tracking publications, identifying bibliometric tools and resources, and investigating methods to use publication data for evaluating their progress. In addition, to assess the scope and impact of cross-disciplinary research collaboration, researchers have applied the theories and models of social network analysis (SNA) to analyze research grant data or grant and publication data together [18–23]. The adopted methods in previous studies focused on measuring the centrality of individual researcher or group in the cross-disciplinary research collaborations, the density of a research network, the strength of the collaborative relationships, the potential to bridge basic science researchers with clinical investigators, the detection of research communities, and the change of collaboration patterns. SNA using bibliographic data, known as bibliometric network analysis, offers many more options for evaluating the development of collaborative research efforts over time – including the geographic dispersion of authors, co-authorship by those in different disciplines, and levels of institutional collaboration [7]. The bibliometric network analysis provides a more comprehensive view of research collaboration at the author, organization, and country levels. Accordingly, specialized bibliometric network analysis tools have been developed (e.g., Sci2, VOSviewer, and CiteSpace) [24–26].

These improved bibliometric measures and network analysis tools not only support the quantitative counting of research outputs but also provide insights regarding the outcomes of the research enterprise and impact of collaboration within and outside of the hub. However, the state-of-the-art bibliometric tools are rarely tested, described, or applied to CTSA research impact evaluations. Therefore, the overall goal of this pilot study was to understand if integrating both improved bibliometric measures and network analysis could complement and lead to a more robust evaluation of the CTSA research outcomes and collaboration impact. Specially, this study focused on North Carolina Translational and Clinical Sciences Institute (NC TraCS), one of the national CTSA hubs at the University of North Carolina at Chapel Hill (UNC-CH) and attempted to address the following evaluation aims:

1. Measure and describe the performance of NC TraCS in supporting the translational and clinical research enterprise at UNC-CH;
2. Assess how much and what forms of interdisciplinary collaborations are catalyzed by NC TraCS-supported research at UNC-CH;
3. Evaluate the extent to which NC TraCS-supported research covers the full continuum of translational research at UNC-CH.

This pilot study was conducted in the context of the Developmental Evaluation and the Context Input Process Product Models [27,28] that NC TraCS uses to guide their overall evaluation approach. It did not use any data to compare the research impact of NC TraCS-supported publications with other CTSAs. Instead, we aimed to bring new evaluation methods, tools, and perspectives to CTSA evaluation through assembling a picture of NC TraCS research outcomes by using selected measures from the advanced and improved bibliometric resources and tools that are currently available to CTSA institutes.

## Methods

### Data Sample

We included publication records from the inception of NC TraCS, September 1, 2008, through March 27, 2017. This study included 754 publications in which the authors cited the NC TraCS grant as supporting their research. These publications reflect a variety of NC TraCS-relevant activities including, but not limited to consultation services, informatics and data science support, study recruitment and coordination assistance, community engagement, pilot awards, training, and career development. The study team downloaded bibliographic records from NC TraCS My NCBI account (Supplementary File 1) and PubMed in Medline format and produced the master data file for analysis.

### Data Tools

The following are the tools used in this pilot study:

- *Scopus*: is one of Elsevier research intelligence products – a citation-tracking database of peer-reviewed literatures. Compared with Web of Science (WoS), it has much broader coverage in biomedical and life sciences [29]. Besides citation counts, Scopus offers two comparative citation impact metrics, Field-Weighted Citation Impact (FWCI) and Citation Benchmarking (CB) (defined below) with a subscription.
- *iCite and NIH Relative Citation Ratio (RCR)*: RCR, a metric generated by iCite, is a new article-level and field-independent metric, measuring an article’s citation impact relative to other NIH-supported research produced in the same field during the same timeframe [30]. It is a validated method to quantify the influence of a research article [31]. With a customizable benchmarking feature, RCRs can be used to compare research performance across different fields and research groups [16,30].
- *VOSviewer (Version 1.6.8)*: is a free bibliometric network analysis software, which was designed to analyze and visualize co-citation, bibliographic coupling, co-authorship relation, and organization networks [25]. It also offers text mining functionality that is used to construct co-occurrence networks for research topics. After evaluating several bibliometric network analysis and visualization tools including Sci2, VOSviewer, and CiteSpace, the researchers for this study chose VOSviewer as the major tool for bibliometric data mining, network construction, and visualization.

### Data Measures

After checking the availability, utility, and interpretability of the metrics offered by the identified tools and other CTSA bibliometric evaluation reports, the study team chose the metrics listed in Table 1. Compared to the traditional bibliometrics measures like the total number of publications or citation counts, comparative citation impact is relatively new, demonstrating a publication’s research influence by comparing it to similar publications [32]. This study chose the FWCI and CB from the Scopus database to compare the citation impact of NC TraCS-supported publications with the world average and chose the RCR to compare the citation influence with similar NIH-funded publications. Scopus database defines similar publications as those that have the same publication year, publication type, and discipline. In addition, Elsevier took a “publication-driven assignment” to calculate the expected citations for similar publications, which allows a publication to be allocated to more than one subcategory so that interdisciplinary works can be accommodated. The FWCI and CB from Scopus indicate comparative citation impact at the article level, showing how citations received by an article compare with the average for similar articles. For example, a FWCI of 1 means the paper has been cited at the world average for similar papers; a FWCI of 1.5 indicates that the paper has been cited 50% more times than expected. Similarly, a CB of 99^th^ percentile is high, which indicates an article is in the top 1% globally [14].

**Table 1.** Overview of data measures

Data measures	Categories	Metrics
Bibliometric measure	Research productivity	Total number of publications in a calendar year
	Citation impact	Total citation counts Average cites per year = total citation counts/total number of publications of a year [14]
	Comparative citation impact	FWCI CB RCR
Collaboration measure	Researchers Organizations TraCS with other CTSAs Countries	Co-authorship network Unit collaboration network TraCS-CTSA collaboration network Country collaboration network
Topic measure	Topic clustering and distribution	Co-occurrence key term mapping

TraCS, Translational and Clinical Science Institute; CTSA, Clinical and Translational Science Award; FWCI, Field-Weighted Citation Impact; CB, Citation Benchmarking; RCR, Relative Citation Ratio

For the RCR calculation, researchers defined the “field” of similar publications using an article’s co-citation networks, which include all the articles that have been cited along with this article by other works. The argument is that the co-citation networks more precisely reflect the interdisciplinary nature of biomedical and health science research, grow over time, and are more flexible and accurate than the traditional bibliometric subject categories such as pharmacology and pharmacy or cell biology [31]. NIH-funded papers are the benchmark for RCRs. For example, “any paper with an RCR 1.0 has an RCR higher than 50% of NIH funded papers” [33] in its field, which means the average citation impact; “a paper with an RCR of 2.0 has received twice as many cites per year as the average NIH-funded paper in its field” [30], indicating a higher-than-average citation impact. The research collaboration measures in this pilot study include a series of extracted collaboration networks, i.e., top productive researchers’ co-authorship network, country collaboration network, internal vs. external organization collaboration networks, and the collaboration network between NC TraCS with other CTSA institutes. Further, the study team analyzed and compared the co-authorship networks of top productive researchers supported by NC TraCS in three distinct 30-month time periods (midway of each NC TraCS CTSA grant cycle).

For this study, a network consists of nodes and edges. A node is the object of interest. For example, in co-authorship networks, each node represents an author, with node size representing the number of publications generated by the author. The color represents different organization clusters or communities that the researcher is affiliated with. An edge is a connection or a relation between two nodes. In the co-authorship networks, each edge between two researchers shows their co-authorship activity. Each edge has a strength, represented by a positive numerical value. The higher the value, the stronger the link. The strength of an edge indicates the number of publications two researchers co-authored in the co-authorship network, the number of publications two organizations collaborated in the organization collaboration network, or the number of publications resulting from collaborations across two countries in the country collaboration network. A research topic map was created by key terms extracted from the title and abstract fields of NC TraCS-supported publications that occurred across papers. Each node is a key term, and each edge indicates that two key terms occur together in a publication. The clusters or communities of the key terms indicate a landscape of translational research phases.

The study team exported citation data of 754 publications from PubMed and matched in Scopus using the PMID, digital object identifier, and title fields. The study team also imported all PMIDs to iCite, and the resulting RCR scores were downloaded for analyses. Of the total 754 publications, 734 (97%) matched in Scopus. All matched publications were exported in full citation record format. In addition, AutoIT (Version v3.3.14.2) [34] was used to automatically scrape the values of FWCI and CB from Scopus for each matched publication. Excel and OpenRefine were used to clean and standardize the exported citation records from PubMed, Scopus, and iCite, involving the following fields, publication year, citation counts, FWCIs, CBs, RCRs, authors, and affiliations. Particularly, to prepare for research collaboration analysis, the variations of an author name or an institution name need to be standardized before quantitative analysis and network visualization. Therefore, exported data were prepared and processed by a combination of functions in Excel (e.g., conditional formatting), Openrefine (e.g., facets/filter), and VOSviewer (e.g., thesaurus), which then were analyzed quantitatively and imported back to VOSviewer to generate graphic representations of collaboration networks and a research topic map using the Create-Map wizard [25].

In addition, due to the completeness and data quality of PubMed citations in the fields of publication year, authors, title, and abstract, the study team decided to use PubMed records (*N* = 754) in Medline format to analyze research productivity, researchers’ co-authorship networks (i.e., collaboration network for top-productive (most published) researchers, co-authorship of most published researchers in each 30 month), and research topic clustering and distribution. Providing citation counts and complete author-affiliation information, Scopus records (*N* = 734) were used to generate the analysis and visualization for citation impact, unit collaboration network (i.e., internal and external organization collaboration networks), TraCS-CTSA collaboration network, and country collaboration network. The analysis for comparative citation impact adopted both Scopus (*N* = 734) and iCite data.

## Results

### Descriptive Statistics

#### Research productivity and citation impact

**Fig. 1.**
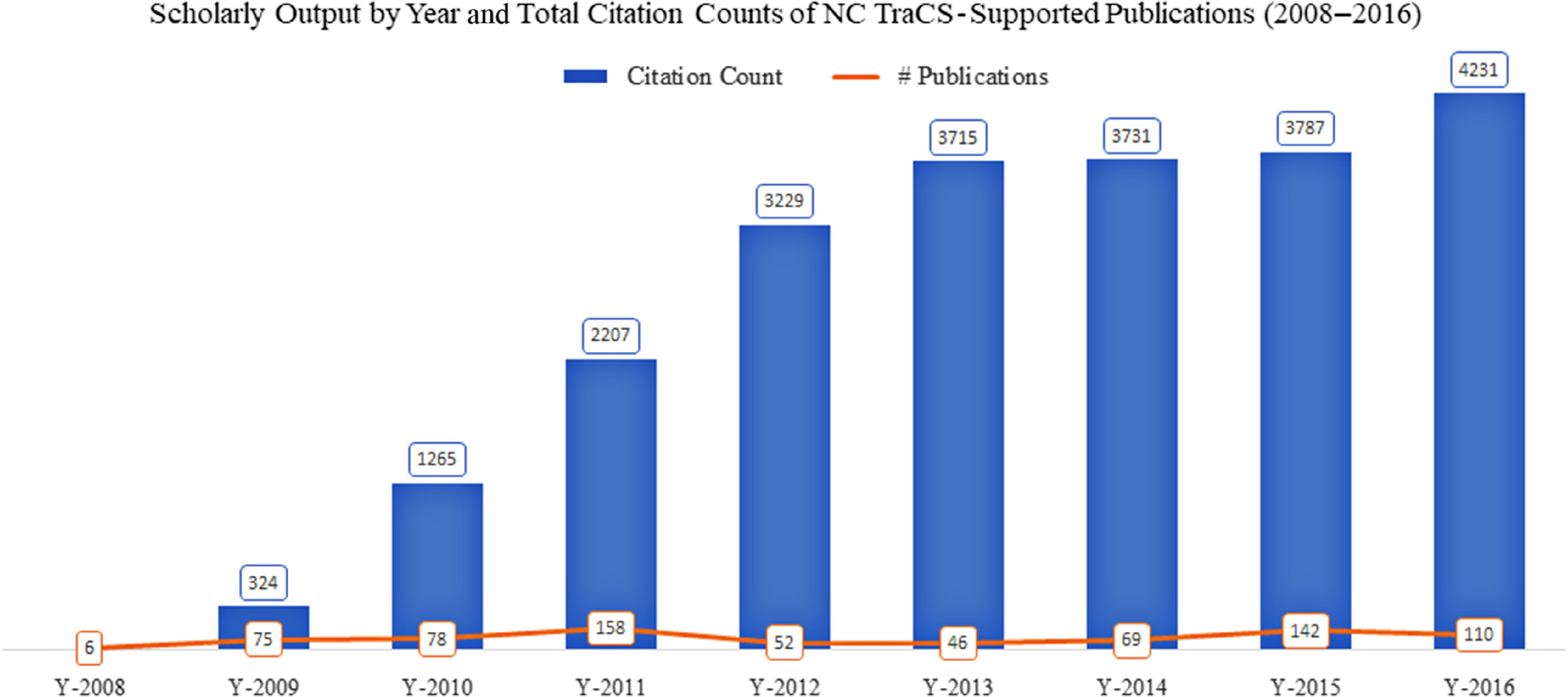
Scholarly output by year and total citation counts of NC TraCS-supported publications (2008–2016) (*N* = 736).

*Note*: the scholarly output of the year 2017 (*n* = 18) was excluded from Fig. 1 because it represented only partial output of this year, i.e., January to March; the citation counts were based on 734 matched Scopus records; and the year of 2017 was also excluded.

The annual research output trend and total citation counts for NC TraCS-supported publications are depicted in Fig. 1. On average, NC TraCS-supported researchers published 82 scientific articles annually from 2008 to 2016. In addition, the total citation counts of NC TraCS-supported papers reached 24,010 by April 20, 2017 (*N* = 734 publications Scopus-matched). A majority (*n* = 12, 80%) of the top 15 most cited articles were published in the program’s first years, i.e., 2008–2010. This is unsurprising given that citations accrue over time. Averagely, each NC TraCS-supported publication was cited 33 times.

**Fig. 2.**
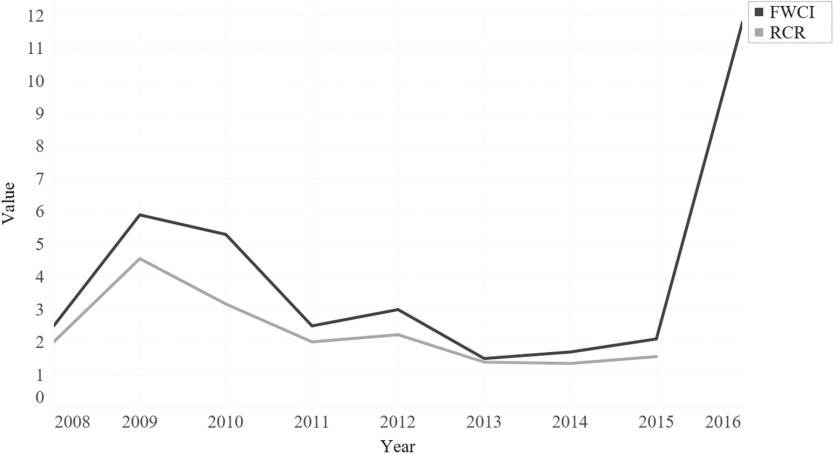
Field-Weighted Citation Impact (FWCI) and Relative Citation Ratio (RCR) distribution of NC TraCS-supported publications.

*Note*: Of the total 754 publications, 734 (97%) matched in Scopus. AutoIT (Version v3.3.14.2) was used to automatically scrape the values of Field-Weighted Citation Impact (FWCI) from Scopus for each matched publication. In addition, the PMIDs of the 754 publications were imported to iCite, and the resulting RCRs were used for annual distribution comparison with FWCIs. The RCRs for the year 2016 were not available yet when this study was being conducted.

Of 754 NC TraCS-supported publications, 752 (99.7%) papers matched with iCite. The mean RCR score of NC TraCS-supported research papers is 2.26, which indicates that NC TraCS-supported publications received more than twice as many cites per year as the average NIH-funded papers in the same field. Fig. 2 shows the annual distribution comparison of both FWCIs and RCRs. Two distribution patterns approximately matched each other. The spike in the 2016 FWCI is driven by an outlier – one highly cited study published with support from NC TraCS [35].

**Fig. 3.**
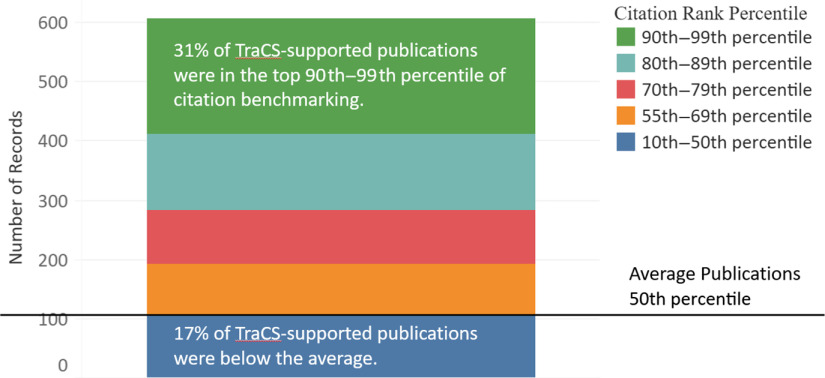
Citation benchmarking of NC TraCS-supported publications (2008–2017).

*Note*: Of the total 754 publications, 734 (97%) matched in Scopus. AutoIT (Version v3.3.14.2) was used to automatically scrape the values of Citation Benchmarking (CB) from Scopus for each matched publication. CB percentiles were available for 607 matched publications when the data were collected in March 2017.

At the time of analysis for this pilot study, Scopus-matched CB data were available for only 607 TraCS-supported publications. Since citation counts need time to accumulate and the CB compares articles within an 18-month window, the CB percentile is usually not available for the articles that are published recently or do not have enough data from similar articles to be compared with. The CB analysis revealed that more than 80% TraCS-supported publications were cited higher than the average publications from similar field (>55th percentile). In addition, 31% of NC TraCS-supported publications were in the top 10% citation rank globally (see Fig. 3).

### Publication Outcomes

#### Research collaborations

**Fig. 4.**
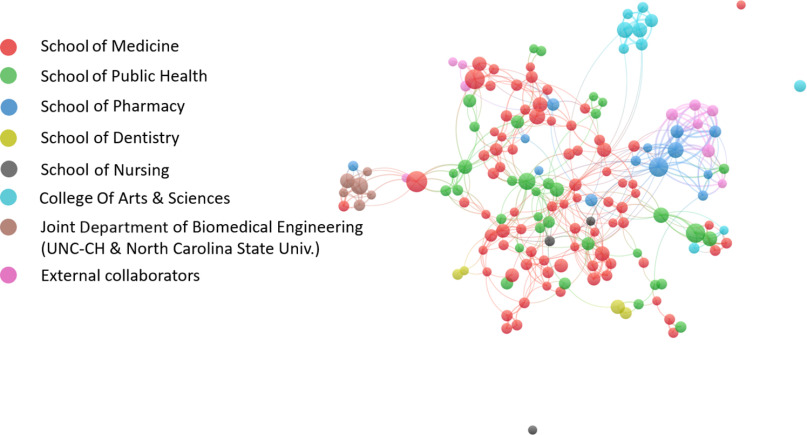
Top productive (most published) researchers’ co-authorship network.

*Note*: Each node represents one author who published five or more articles from 2008 to 2017. The node size corresponds to the number of publications generated by the author. The color represents different organization clusters that the author is affiliated with. An edge is a connection between two nodes. Each edge between two authors shows their co-authorship activity.

To construct co-authorship networks, more than 3000 unique author names were extracted from NC TraCS-supported publications (*N* = 754). Among them, 156 authors contributed to at least five publications. Fig. 4 shows the co-authorship collaboration network of these 156 top-productive (most published) researchers.

**Fig. 5.**
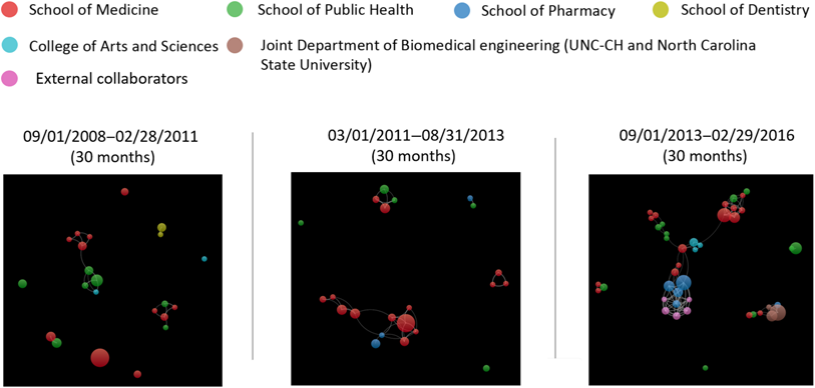
Co-authorship of most published researchers in each 30-month period.

*Note*: Each node represents one author who published five or more articles in each 30-month period. The node size corresponds to the number of publications generated by the author. The color represents different organization clusters that the author is affiliated with. An edge is a connection between two nodes. Each edge between two authors shows their co-authorship activity. The three top productive researchers affiliated with the UNC School of Nursing in Fig. 4 were excluded in 30-month analysis because none of them produced 5 or more papers in each 30-month period although they produced 5 or more publications during the overall examined period 2008–2017.

These authors are mostly from seven academic units at UNC-CH as indicated by the colors of the nodes (i.e., UNC School of Medicine, Public Health, Pharmacy, Dentistry, and Nursing, College of Arts & Sciences, and UNC-North Carolina State University Joint Department of Biomedical Engineering). Within this top researcher collaboration network in which each node/author has at least 5 publications, the average number of co-authors/collaborators of each node/author increased from two in the initial 30 months (September 1, 2008–February 28, 2011) to three in the second 30 months (March 1, 2011–August 31, 2013) and five in the last 30 months (September 1, 2013–February 29, 2016) (Fig. 5). In addition, the average number of co-authored publications by identified top productive researchers grew within each 30-month time period examined: 6 in the initial 30 months; 8 in the second 30 months; 19 in the last 30 months. Moreover, the number of top productive researchers increased over the reporting period (i.e., 22 in the initial 30 months to 43 in the last 30 months), and they demonstrated a denser and more cross-disciplinary collaboration network, indicated by the growing number of participating researchers and the variety of their affiliations.

The construction of an internal collaboration network focused on the most granular UNC units such as departments, centers, and divisions. Data on more than 300 unique UNC units were extracted and showed 95% of the units represented all eight schools at UNC – Medicine, Public Health, Pharmacy, Dentistry, Nursing, Social Work, Information & Library Science, and Arts & Science (*N* = 734 Scopus-matched publications). Particularly, UNC Department of medicine, biostatistics, and epidemiology were the top three most collaborative UNC units.

**Fig. 6.**
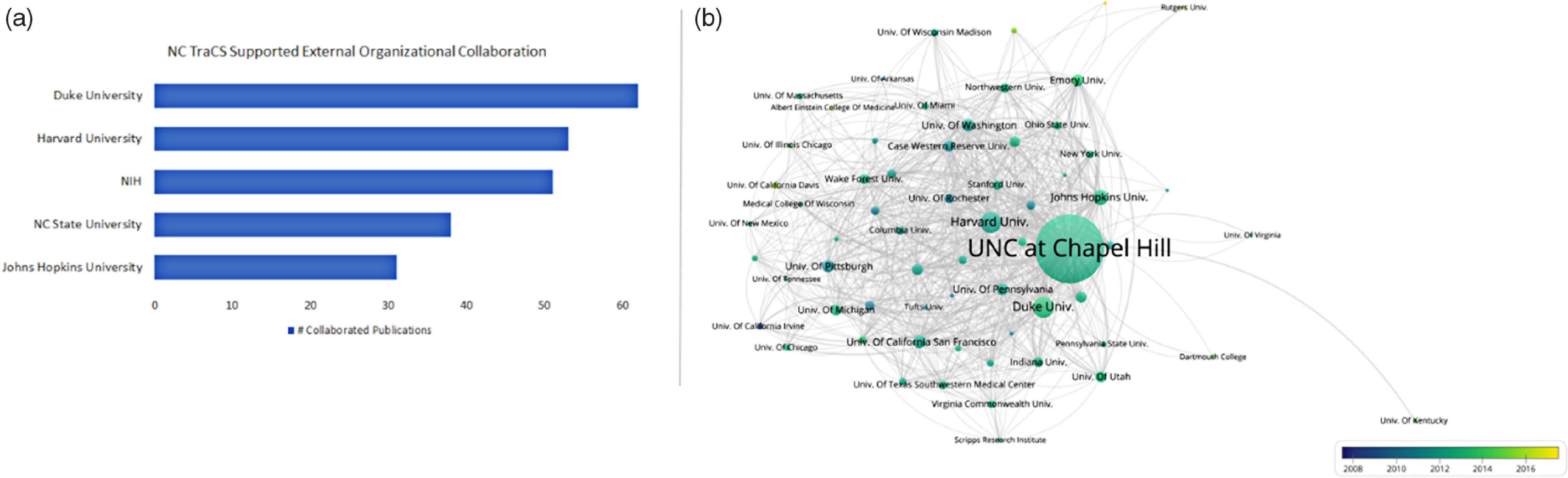
(a) Top external collaborated institutions of NC TraCS-supported research; (b) collaboration network of NC TraCS-supported research with other CTSA institutes (*n* = 65).

*Note*: In (b), each node represents one institute that TraCS-supported researchers collaborated with. The node size corresponds to the number of publications generated by the institute. The color represents time (2008–2017). An edge is a connection between two nodes. Each edge between two institutes shows their co-authorship activity.

The external organization collaboration network focused on the units that are outside of UNC-CH at the macro level. Collaboration across institutions is now a norm in biomedical research, and 56% of the publications analyzed included co-authors from other institutions. Approximately 800 unique external organizations were identified through author affiliation data analysis. The top external organizational collaborators were Duke University (including Duke Clinical Research Institute, Duke Medical Center, and Duke-National University of Singapore Graduate Medical School), Harvard University (including Harvard Medical School, Harvard School of Public Health, Broad Institute of MIT and Harvard), NIH (including National Cancer Institute, National Institutes of Diabetes and Digestive and Kidney Diseases, National Institute of Allergy & Infectious Diseases, and National Institute of Environmental Health Sciences), NC State University (UNC-NC State University joint department of Biomedical Engineering, College of Veterinary Medicine, Department of Statistics, Department of Entomology, etc.), and Johns Hopkins University (Fig. 6a).

NC TraCS-supported researchers continue to collaborate with their colleagues at other CTSA hubs to accelerate and catalyze clinical and translational research in the USA. Specifically, approximately half (*N* = 302, 41%) of the publications were generated in collaboration with researchers at other CTSA hub institutes (Fig. 6b). While the CTSA consortium primarily focuses on clinical and translational research conducted within the USA, the findings showed that collaborations occurred worldwide. The country collaboration network shows NC TraCS-supported researchers collaborated with other researchers from 48 countries. About 20% of publications included co-authors from different countries. United Kingdom, Canada, China, Malawi, and Germany accounted for almost three-fourths (72%) of those collaborations.

#### Research topic mapping

Three hundred and five key terms (i.e., occur more than 10 times across all publications) were extracted from both the titles and abstracts of NC TraCS-supported publications to construct a topic map. These key terms formed four clusters revealing a research landscape from clinical/lab/disease, therapy/medication, provider/care/practice to race/sex/population. This topic map demonstrates that NC TraCS-supported research has focused on all phases of translational research, i.e., “bench to population” [36].

## Discussion

Our CTSA case study is the first to highlight the complementary role of applying both improved bibliometric measures and an advanced bibliometric network analysis tool to evaluate research impact; we used NC TraCS, a CTSA, as a case study of these methods. First, NC TraCS-supported research has produced impactful scientific output since its inception in 2008. Despite the great possibility of being underestimated, a total of 754 publications were identified by their authors as receiving support from NC TraCS, resulting in an average of over seven articles per month. These articles have accumulated over 24,000 citation counts by April 2017 and on average, each article has received 33 cites to date according to the data provided by Scopus, one of the authoritative citation-tracking databases in the world. The distribution patterns between FWCI and RCR approximately matched which are consistent with the finding of a strong correlation between FWCI and RCR from a recent study [37]. In addition, NC TraCS-supported research papers received more than twice as many cites per year as the average NIH-funded papers or the world average for similar papers, indicating the citation impact of NC TraCS-supported research.

Second, bibliometric network analysis looked at three dimensions of collaboration in publications: co-authorship across disciplines; organizational collaborations identified in the articles, both within and outside UNC-CH; and the geographic distribution of collaborations. Notable results from this analysis include (1) the co-author collaboration network helped NC TraCS identify the top productive researchers and their collaboration communities. The most prolific researchers formed a dense multidisciplinary collaboration network which involved seven major academic units and external partners. In addition, within the co-authorship network of top productive researchers, the average number of co-authors (who are also most published researchers) of each identified top author increased from two in 2008 to five by 2016, indicating that NC TraCS’ support significantly contributed to increased researchers’ collaboration network and their research productivity. NC TraCS continues to create and facilitate opportunities for interdisciplinary collaboration and connecting researchers with right peers and organizations. (2) More than half of the analyzed publications included co-authors from other institutions. In addition, approximately half of NC TraCS-supported publications were results of collaboration with other CTSA institutes in the consortium, confirming NIH’s goal of broad consortium collaboration. (3) About 20% of all publications included co-authors working in countries other than the USA, again suggesting opportunities for new insights.

Third, the research topic network created by the bibliometric network analysis and visualization tool identified major areas of NC TraCS-supported research including, but not limited to lab discovery, clinical research, physician care, and population science. This identification demonstrated that NC TraCS continues to support the mission of NCATS. The topic mapping metrics of NC TraCS-supported publications go beyond the scope of bibliometrics by measuring the foci of translational science activities at an institution [16,38].

Although citation tracking and analysis provided as part of the services of Scopus, WoS, and other citation databases can directly answer questions about publication output and suggest latent impact, several complications emerge when attempting to put the citation metrics into context. First, without a meaningful comparison, there is no basis for assessing this metric as representing relative success/failure in the generation of scientific output [4]. Second, publication counts offer a very limited reflection of scientific output. For instance, in 2017, a component of NC TraCS that helped researchers access biomedical informatics tools and developed appropriate approaches worked on 700 researcher requests. While this service advanced those studies, many of the researchers may have failed to cite NC TraCS as a supporter in their publications. Third, although bibliometric measures can delineate collaboration activities at the behavior level, additional methods and studies will be needed to help identify factors other than CTSAs which contributed to the collaboration patterns.

NC TraCS received approximately 2500 requests for research services in the grant year 2016–2017. Grant citations reflect the diligence of NC TraCS in requesting that researchers cite them, the determination of the author to do so, and whether the publication is indexed by Scopus or PubMed. For these reasons, the publication count can suggest that scientific output is occurring but does not accurately or contextually reflect the full impact of a CTSA on translational science research output.

Adopted by several previous CTSA-related bibliometric studies [4,16,17,39], RCR, provided by the NIH through iCite, is a useful metric for getting a sense of the impact of published research, informing the respective CTSA leadership that CTSA-supported publications are influencing other researchers. Our future research will stratify the publications by citation impact percentiles and see how the percentages in each percentile shift: (1) over time; (2) depending on the types of NC TraCS services utilized; and (3) depending on the extent to which the researcher worked with NC TraCS in the development of their research (dosage).

Our pilot study highlights the limitations to this bibliometrics approach. First, the publications included in this study are only a fraction of the scientific output of TraCS-supported research due to underestimated grant citations in publications and limited indexing scope of citation databases. Thus, the results reflect only partial impact of TraCS-supported translational research output. Second, each institution must rely on their own knowledge, preference, and access to bibliometric resources and tools for research impact assessment. Therefore, the reported outcomes could come from different resources and are hard to be compared and benchmarked across institutions. For example, Scopus, WoS, and iCite all provide citation counts and comparative citation indicators. However, they have different journal coverage and algorithms to categorize subject areas. Not only does the citation count vary for one article but also the top percentile publications in one database may not be the top in another. In addition, UNC-CH subscribed to SciVal [14] in the past to provide the FWCI and CB across institutions. Since UNC-CH discontinued the subscription in early 2016, researchers had to adopt alternative methods to retrieve the FWCI and CB at the article level. However, the citation impact benchmarking across institutions becomes impossible due to lack of access to the aggregated metrics data that SciVal provides. Third, some CTSAs have longer history and variable award sizes. Since the research output productivity depends on the funding scale and citation counts build up over time, the bibliometric comparison across CTSAs can be biased.

## Conclusion

This pilot study adopted bibliometrics and bibliometric network analysis to measure the research output and collaboration impact of a CTSA program hub. Both the traditional measures (e.g., publication and citation count) and the improved new field- and time-normalized measures developed by Scopus and NIH iCite, i.e., FWCI, CB, and RCR, were applied to evaluate NC TraCS-supported publications from 2008 to 2017. We were encouraged that collaboration seemed to increase over time, supporting the impact of the CTSA consortium on the conduct of translational research. This study is one of the few to go beyond the use of traditional reporting measures to construct bibliometrics collaboration networks to assess research impact of CTSA-supported publications using a specialized bibliographic data analysis tool. Bibliometric assessment of scientific research impact is starting to gain favor in CTSA hubs. However, there is no standard set of bibliometric measures and reporting format within the CTSA consortium [4]. Although the bibliometrics approach is a valid method to assess research impact, it still needs to be customized and improved to suit CTSA evaluation needs.

## Supplementary material

For supplementary material accompanying this paper visit https://doi.org/10.1017/cts.2020.29. click here to view supplementary material
