# Supplementary material for: Bibliometrics approach to evaluating the research impact of CTSAs: A pilot study
Source: J Clin Transl Sci. 2020 Apr 2;4(4):336–44. doi: 10.1017/cts.2020.29 (PMC7681148; doi:10.1017/cts.2020.29)
Supplement: Supplementary file 1 [file S2059866120000291sup001.docx]

**APPENDIX**

**Saved Search Queries in TraCS My NCBI Account**

**Search query #1**

((TTSA*[Grant Number] OR "nc tracs"[All Fields] OR "nc translational"[All Fields] OR "north carolina translational"[All Fields])) NOT (UL1TR002489 OR KL2TR002490 OR TL1TR002491 OR 1UL1TR001111 OR UL1TR001111 OR 1UL1TR001111-01 OR 1UL1TR001111-02 OR 1UL1TR001111-03 OR 1UL1TR001111-04 OR 1UL1TR001111-05 OR UL1TR001111-01 OR UL1TR001111-02 OR UL1TR001111-03 OR UL1TR001111-04 OR UL1TR001111-05 OR 1KL2TR001109 OR KL2TR001109 OR 1KL2TR001109-01 OR 1KL2TR001109-02 OR 1KL2TR001109-03 OR 1KL2TR001109-04 OR 1KL2TR001109-05 OR KL2TR001109-01 OR KL2TR001109-02 OR KL2TR001109-03 OR KL2TR001109-04 OR KL2TR001109-05 OR 1TL1TR001110 OR TL1TR001110 OR 1TL1TR001110-01 OR 1TL1TR001110-02 OR 1TL1TR001110-03 OR 1TL1TR001110-04 OR 1TL1TR001110-05 OR TL1TR001110-01 OR TL1TR001110-02 OR TL1TR001110-03 OR TL1TR001110-04 OR TL1TR001110-05 OR 5UL1TR000083-05S1 OR UL1TR000083-05S1 OR 5UL1TR000083 OR UL1TR000083 OR UL1TR000083 OR KL2TR000084 OR TL1TR000085 OR UL1RR025747 OR KL2RR025746 OR TLRR025745 OR U54RR024383 OR M01RR00046)

**Search query #2**

UL1TR002489 OR KL2TR002490 OR TL1TR002491 OR 1UL1TR001111 OR UL1TR001111 OR 1UL1TR001111-01 OR 1UL1TR001111-02 OR 1UL1TR001111-03 OR 1UL1TR001111-04 OR 1UL1TR001111-05 OR UL1TR001111-01 OR UL1TR001111-02 OR UL1TR001111-03 OR UL1TR001111-04 OR UL1TR001111-05 OR 1KL2TR001109 OR KL2TR001109 OR 1KL2TR001109-01 OR 1KL2TR001109-02 OR 1KL2TR001109-03 OR 1KL2TR001109-04 OR 1KL2TR001109-05 OR KL2TR001109-01 OR KL2TR001109-02 OR KL2TR001109-03 OR KL2TR001109-04 OR KL2TR001109-05 OR 1TL1TR001110 OR TL1TR001110 OR 1TL1TR001110-01 OR 1TL1TR001110-02 OR 1TL1TR001110-03 OR 1TL1TR001110-04 OR 1TL1TR001110-05 OR TL1TR001110-01 OR TL1TR001110-02 OR TL1TR001110-03 OR TL1TR001110-04 OR TL1TR001110-05 OR 5UL1TR000083-05S1 OR UL1TR000083-05S1 OR 5UL1TR000083 OR UL1TR000083 OR UL1TR000083 OR KL2TR000084 OR TL1TR000085 OR UL1RR025747 OR KL2RR025746 OR TLRR025745 OR U54RR024383 OR M01RR00046
